# Supplementary material for: The effect of an additional pre-extubational loading dose of caffeine citrate on mechanically ventilated preterm infants (NEOKOFF trial): Study protocol for a multicenter randomized clinical trial
Source: PLoS One. 2025 Jan 13;20(1):e0315856. doi: 10.1371/journal.pone.0315856 (PMC11730378; doi:10.1371/journal.pone.0315856)
Supplement: S2 File — (PDF) [file pone.0315856.s002.pdf]

INFORMATION SHEET

FOR PARENTS/LEGAL GUARDIAN

Title: The effect of an additional pre-extubational loading dose of caffeine citrate on mechanically ventilated preterm infants (Randomized Clinical Trial)

Protocol number of the trial: NEOKOFF22

Principal investigator: Ákos Gasparics MD. Ph.D., Semmelweis University, Department of Obstetrics and Gynecology, Baross Street Branch, Budapest, Baross Street 27. 1088

Dear Parents/Guardians,

We kindly request that you consider participating in this study only after thoroughly reviewing this information sheet and engaging in a comprehensive discussion with the investigating physician. It is essential that you take the time to fully comprehend the details of what is being proposed.

<u>Purpose and Procedure of the Research:</u>

Apnea of prematurity (AOP)

Apneas occur due to the immature central nervous system, the immature respiratory center of premature infants, which can be accompanied by decreasing pulse rates. These apneas arise more frequently and are more severe in premature infants with lower gestational ages. Before the 28th week of pregnancy, or under 1000 grams of birth weight, almost all premature infants are affected. We continuously monitor premature infants' blood oxygen levels and pulse rates in our department.

Mechanical Ventilation, Extubation

During the handling of premature infants, mechanical ventilation might be necessary in some cases for various reasons, such as frequent apneas. During this process, a thin tube is inserted into the trachea, allowing the ventilator machine to perform partial or all breathing works

instead of the premature infant. Mechanical ventilation can have numerous complications, so our goal during treatment is to minimize the time spent on the ventilator machine.

As soon as the condition of the premature infant allows, we remove the tube (extubation). After extubation, the premature infant performs every breath, and most of the respiratory work is carried out by themselves, assisted by non-invasive respiratory support (CPAP, DUOPAP). Extubation is unsuccessful in every case, and mechanical ventilation is applied again.

Caffeine Treatment

Caffeine treatment begins after birth in the case of all infants born before the 32nd week of pregnancy. The administration of caffeine at neonatal intensive care units has become widespread worldwide after it has been proven to reduce the frequency of apneas and the development of chronic lung disease. It promotes weaning from the ventilator, successful extubation, and improves survival without developmental neurological complications. Side effects of caffeine treatment may include increased heart rate, reduced gastrointestinal motility, high blood pressure, and neurological abnormalities (e.g., irritability and tremors).

We use caffeine according to the most commonly used dosing regimen worldwide. After birth, premature infants receive a higher, so-called "loading dose" on the first day of life. After this, they receive caffeine in a lower, so-called "maintenance dose" once or twice daily, depending on the frequency of apneas.

Application of a Single Loading Dose of Caffeine Citrate Before Extubation

The beneficial effects of caffeine are known, but the "optimal" dosing regimen is not clear. Extubation failure and prolonged mechanical ventilation increase the frequency of acute and chronic complications; therefore, we aim to increase the number of successful extubations as much as possible. Because of these, we modify the caffeine dosing regimen in one group of mechanically ventilated premature infants participating in the study: instead of the lower "maintenance dose," we administer a higher "loading dose" of caffeine before extubation.

Based on laboratory measurements supported by literature data, a higher dose of caffeine can be safely administered, but the rate of side effects might be elevated.

A total of 226 premature infants are planned to be included in the study. Intervention and participation in the study do not involve additional blood collection or pain for children. Premature infants participating in the study will be followed up to age two with regular (every six months) developmental neurological examinations.

Data recording, Database

Clinical data are recorded during the treatment of premature infants, and bedside examinations (blood glucose measurement, blood gas analysis) are also performed. If you consent, we will store the clinical data collected during intensive treatment coded in a database.

The data will not contain personal information. They are recorded anonymously, coded, and can only be accessed by the personnel conducting the study and the supervisory authority. Copies of this data cannot be made.

Information relating to pregnancy, data generated during the neonatal intensive care unit stay, and information collected during follow-up examinations will be necessary for the study. The scientific evaluation of the data helps to improve the effectiveness of the medical treatment of premature infants.

Data Management

The current legal regulations applicable to data protection are the Regulation (EU) 2016/679 of the European Parlament and of the Council on the protection of natural persons with regard to the processing of personal data and on the free movement of such data, repealing Directive 95/46/EC (General Data Protection Regulation) on the European level, Act CXII of 2011 on the right to informational self-determination and the freedom of information, with legislative amendment in effect from July 1, 2018 and Act XLVII of 1997 on the Processing and Protection of Medical and Other Related Personal Data on the national level. The data controller for the data collected in the study is Semmelweis University. The university's data

protection officer is Sára Trócsányi MD. Ph.D., email: adatvedelem@semmelweis-univ.hu, Tel.: 061/459-1500, extension: 62710.

If you have any complaints regarding data processing or if your data protection rights have been violated, you can contact the data protection supervisory authority or the court. In Hungary, the data protection supervisory authority is the National Data Protection and Freedom of Information Authority (Budapest, Falk Miksa Street. 9-11 1055; phone: 06-1-391-1400, email: ugyfelszolgalat@naih.hu, website: www.naih.hu).

Possible and Expected Consequences

Data breaches may occur during data processing, resulting in compensation for the participant.

Way to Mitigate Risks and Potential Damage

Considering that your child will not receive experimental treatment and there will be no examination that is not already known in the field of neonatology, there are no specific risks associated with participation. The Patient Rights Representative of your treatment facility (name: Dr. Anikó Gachályi, Patient Rights Representative. Contact: +36 20 4899 525; email: aniko.erhardtne@ijb.emmi.gov.hu) can assist with questions that arise during routine patient care.

Insurance for the Study: Semmelweis University has liability insurance with Allianz Hungária Zrt.

Who Reviewed and Approved the Study?

The Health Science Council's Clinical Pharmacology Ethics Committee (ETT KFEB) provided a professional and ethical opinion. The study is conducted based on the approval of the National Institute of Pharmacy and Nutrition (OGYEI).

Approval number: OGYÉI/6838-11/2023

Participation in the Study

If you decide not to participate in the study, your child will still receive the most careful intensive therapy.

If you choose to participate in the study, your child will be randomly assigned to either the control group (routine dosage) or the group treated with a higher dose of caffeine planned before extubation. The examinations and data recording will be carried out in the same way in both groups.

If you have decided to participate in the study, you have the right to withdraw your consent at any time, verbally or in writing, without providing a reason. Your decision will not in any way affect your child's medical care. Please feel free to approach the staff caring for your child to facilitate your decision-making process. It is important to emphasize that neither our research team nor the medical staff has any personal interest in the participation of children in the study.

If any protocol changes during the study, a new patient information sheet and consent form will be presented and signed accordingly.

Compensation: Patients participating in the study will not receive any compensation.

I declare that I have understood the oral and written explanations regarding the study ("The effect of an additional pre-extubational loading dose of caffeine citrate on mechanically ventilated preterm infants), and I acknowledge my responsibility as a participant. I had the opportunity to ask any questions I deemed necessary and received satisfactory answers. The provided information outlined the objectives and methods, expected benefits, possible risks and discomforts, and how and when results will be communicated to me. Furthermore, it was guaranteed that there would be no charges associated with participation in the program, no compensation for participation, and I have the right to refuse participation verbally or in writing at any time without any loss. Additionally, I was informed that the samples and

clinical data, while maintaining anonymity, may be shared with authorized researchers bound by professional confidentiality.

Under the given conditions, I freely consent to allow the person under my supervision to participate in the presented research program. Under the described circumstances, I consent to the provision of clinical data.

I have read and understood the information sheet and received satisfactory answers. I have received a copy of the information sheet.

Budapest,

Signature of Parent/Legal Guardian:

____

Statement of the person obtaining consent (attending physician or researcher): I declare that I have explained the study's essence and the program's objectives to the participant, including the possible benefits and risks, and I have answered all questions to the best of my knowledge. Statement of the person obtaining consent (attending physician or researcher):
Name: ____

Signature: ____

Two copies of the consent form have been prepared. One copy of the document will be handed over to the participant.

INFORMED CONSENT FOR PARENTS/LEGAL GUARDIAN

Title: The effect of an additional pre-extubational loading dose of caffeine citrate on mechanically ventilated preterm infants (Randomized Clinical Trial)

Protocol number of the trial: NEOKOFF22

Participant's Information

Name: ……………………………………………………………………………………

Mother's name: ……………………………………………………………………………….

Date of birth: …………………………………………………………………………………….

Place of birth: …..…………………………………………………………………….…………

Social security number (TAJ): ………………………………………………………………..

Address: ……………………………………………………………………………………………

Legal Guardian's Identification Information:

Name: ……………………………………………………………………………………

Mother's name: ……………………………………………………………………………….

Date of birth: ……………………………………………………………………………….....

Place of birth: ……...………………………………………………………………………….

Social security number (TAJ): ………………………………………………………………..

Address: ……………………………………………………………………………………………

I, (full name) ______________________________________ responsible for (full name) ______________________________________ hereby declare that I have understood the oral and written explanations regarding the study ("The effect of an additional pre-extubational loading dose of caffeine citrate on mechanically ventilated preterm infants ") for which my responsibility as a participant is requested. I had the opportunity to ask any questions I deemed necessary and received satisfactory answers. The provided information outlined the objectives and methods, expected benefits, possible risks, and discomforts, how and when results will be communicated to me and the possibility of future contact. Furthermore, it was guaranteed that there would be no charges associated with participation in the program, no compensation for participation, and I have the right to refuse participation verbally or in

writing at any time without any loss. Additionally, I was informed that the clinical data, while maintaining anonymity, may be shared with authorized researchers bound by professional confidentiality.

Under the given conditions, I freely consent to allow the person under my supervision to participate in the presented research program, and under the described circumstances, I consent to the provision of clinical data and future contact.

Budapest,

Signature of Parent/Legal Guardian:

Statement of the person obtaining consent (attending physician or researcher): I declare that I have explained the study's essence and the program's objectives to the participant, including the possible benefits and risks, and I have answered all questions to the best of my knowledge.

Statement of the person obtaining consent (attending physician or researcher):

Name: ____

Signature:____

Date:______________________

Two copies of the consent form have been prepared. One copy of the document will be handed over to the participant.
