# Supplementary material for: The effect of an additional pre-extubational loading dose of caffeine citrate on mechanically ventilated preterm infants (NEOKOFF trial): Study protocol for a multicenter randomized clinical trial
Source: PLoS One. 2025 Jan 13;20(1):e0315856. doi: 10.1371/journal.pone.0315856 (PMC11730378; doi:10.1371/journal.pone.0315856)
Supplement: S3 File — (PDF) [file pone.0315856.s003.pdf]

SUMMARY OF PRODUCT CHARACTERISTICS

1. NAME OF THE MEDICINAL PRODUCT

CITRATE DE CAFEINE COOPER 25 mg/mL, injectable and oral solution

2. QUALITATIVE AND QUANTITATIVE COMPOSITION

Caffeine citrate ,………………………………………………… 25.0 mg
(equivalent to anhydrous caffeine base………………………… 12.5 mg)
(and anhydrous citric acid…………………………………… 12.5 mg)

Per 1 mL of solution

One ampoule of 2 mL contains 50 mg of caffeine citrate.
For the full list of excipients see section 6.1

3. PHARMACEUTICAL FORM

Injectable and oral solution.

4. CLINICAL PARTICULARS

4.1. Therapeutic indications

Treatment of apnoea in the premature infant.

4.2. Posology and method of administration

Method of administration

For intravenous or oral route.
Do not inject intramuscularly (see section 4.3).

Posology

One ampoule of 2 mL contains 50 mg of caffeine citrate, equivalent to 25 mg of caffeine base.

Loading dose:
20 mg/kg/day of caffeine citrate (equivalent to 10 mg/kg/day of caffeine base) by slow intravenous administration.

Maintenance dose (generally from 24 hours after the loading dose):
5 mg/kg/24 hours of caffeine citrate (equivalent to 2.5 mg/kg/24 hours of caffeine base) orally or by IV infusion.
The prescription must state the dosage in caffeine citrate as this is the dosage expressed for the name of the product.

4.3. Contraindications

- hypersensitivity to the active substance, to xanthine derivatives or to any of the excipients
- IM route.

4.4. Special warnings and precautions for use

Monitoring of blood caffeine concentrations is essential in order to maintain a therapeutic plasma concentration of between 8 and 15 mg/L.

Caffeine should be used with caution in patients with cardiac rhythm disorders, epilepsy or hyperthyroidism.

Combination with other central nervous system stimulants carries a risk of potentiating toxicity (see section 4.5).

Because of its relaxant effect on the oesophago-gastric junction, caffeine may worsen gastro-oesophageal reflux.

As with all pre-term infants, neonates treated with caffeine citrate should be closely monitored for signs suggestive of neonatal necrotising enterocolitis (see section 4.8). A possible association between the use of caffeine citrate and development of necrotising enterocolitis in pre-term infants has been reported. However, no cause/effect relationship has been established between caffeine and neonatal necrotising enterocolitis.

4.5. Interaction with other medicinal products and other forms of interaction

Combinations not recommended

\+ **Enoxacin (reported in adults)**: A large increase in caffeine concentrations in the body which may result in excitation and hallucinations (reduced hepatic metabolism of caffeine).
Risk of caffeine overdose.

Combinations to take into account

\+ **Ciprofloxacin, norfloxacin (reported in adults)**: large increase in caffeine concentrations in the body (reduced hepatic metabolism of caffeine).
Risk of caffeine overdose.

\+ **Cimetidine (in adults and children)**: possible reduction in hepatic metabolism of caffeine caused by cimetidine. The interaction is probably weak in newborn infants because the caffeine is eliminated principally by the kidney.

\+ **Phenytoin**: phenytoin may increase elimination of caffeine.
Clinical and laboratory (blood caffeine concentrations) monitoring is required.

\+ **Beta-2 mimetics**: beta2-mimetics may potentiate the positive inotropic effect of caffeine.
Clinical monitoring is required.

\+ **Other psychostimulants**: central nervous system stimulants may potentiate the neurotoxic effects of caffeine.
Clinical monitoring is required.

\+ **Medicinal products that inhibit gastric acid secretion (histamine H2-receptor blockers or proton-pump inhibitors)**

The inhibition of gastric acid secretion, being associated with bacterial proliferation in the gut, may potentiate the development of neonatal necrotising enterocolitis (see sections 4.4 and 4.8). As with all pre-term infants, neonates should be closely monitored for signs suggestive of neonatal necrotising enterocolitis.

4.6. Fertility, pregnancy and lactation

Not applicable.

4.7. Effects on ability to drive and use machines

Not applicable

4.8. Undesirable effects

Adverse reactions reported with CITRATE DE CAFEINE COOPER 25 mg/mL, oral solution, and solution for injection, are listed below by System Organ Class (SOC) and frequency. Frequency is defined as: very common (≥ 1/10), common (≥1/100 to < 1/10), uncommon (≥1/1,000 to <1/100), rare (≥1/10,000 to <1/1,000), very rare (<1/10,000) and not known (cannot be estimated from the available data).

Tabulated list of adverse reactions

SOC (MedDRA)	Frequency	Adverse reaction
Metabolism and nutrition disorders	Not known	Hyperglycaemia
Psychiatric disorders	Not known	Agitation*, hyperexcitability*
Nervous system disorders	Not known	Trembling*, tremors*
Cardiac disorders	Not known	Tachycardia*
Gastrointestinal disorders	Not known	Vomiting*, diarrhoea*, neonatal necrotising enterocolitis**
Renal and urinary disorders	Not known	Polyuria*
Investigations	Not known	Abnormal catecholamines in urine

* These signs may be suggestive of a possible overdose and plasma caffeine levels monitored accordingly.

** A possible association between the use of caffeine citrate and development of neonatal necrotising enterocolitis in pre-term infants has been reported in the literature. However, no cause/effect relationship has been established between caffeine use and neonatal necrotising enterocolitis. A multicentre study (n=2006) investigating the long-term outcome of pre-term infants treated with caffeine citrate did not observe any increased frequency of neonatal necrotising enterocolitis in the caffeine group when compared to the placebo group. A ten-year case-control study (n=170) concluded that there was no correlation between cumulative doses of caffeine citrate and the development of neonatal necrotising enterocolitis in neonates. As with all pre-term infants, neonates treated with caffeine citrate should be closely monitored for signs suggestive of neonatal necrotising enterocolitis.

Reporting of suspected adverse reactions

Reporting suspected side effects after authorisation of the medicinal product is important. It allows continued monitoring of the benefit/risk balance of the medicinal product. Healthcare professionals are asked to report any suspected adverse reactions via the national reporting system: *Agence Nationale de Sécurité du Médicament et des Produits de Santé* (ANSM, (French National Agency for Medicines and Health Products Safety)) and the network of the Regional Pharmacovigilance Centres - Website: www.signalement-sante.gouv.fr.

4.9. Overdose

Initial symptoms suggestive of overdose:

Plasma caffeine concentrations should be measured. These generally occur at plasma concentrations of over 20 mg/L:

- Gastrointestinal disorders: vomiting, diarrhoea, gastric pain.

- Neuropsychiatric disorders: sleep disturbance, trembling, hyperexcitability, agitation (it should be noted that hallucinations and anxiety are not detectable in new-born infants).

- Tachycardia, increased urine output.

Symptoms suggestive of serious overdose:

These generally occur at plasma concentrations of over 50 mg/L:

- central nervous system stimulation with seizures,
- large increase in urine output, dehydration,
- abdominal pain, gastrointestinal haemorrhage,
- acidosis, hypokalaemia, hyponatremia, hyperglycaemia,
- hypercatabolism, hyperthermia, very rarely rhabdomyolysis,
- cardiac rhythm disorders, very rarely ventricular tachycardia

Symptomatic treatment: resuscitation in a specialised unit.

5. PHARMACOLOGICAL PROPERTIES

5.1. Pharmacodynamic properties

Pharmacotherapeutic group: CENTRAL RESPIRATORY STIMULANT/PSYCHOSTIMULANT, ATC Code: R07AB

Caffeine: methylxanthine, central nervous system and respiratory centre stimulant. Caffeine is an inhibitor of phosphodiesterase which is an enzyme responsible for inactivation of 3', 5' cyclic adenosine monophosphate (cAMP). Increased levels of cAMP within the cell act as the mediator responsible for the main pharmacological actions of caffeine.
Caffeine therefore stimulates the entire central nervous system, and in particular the respiratory centres. It may produce vasoconstriction of the cerebral blood vessels. It has effects on the cardiovascular, respiratory and gastrointestinal system and in addition acts on skeletal muscle, renal blood flow, glycogenolysis and lipolysis.

5.2. Pharmacokinetic properties

Absorption

Caffeine is completely and rapidly absorbed after oral ingestion. Peak concentrations are reached 15 to 45 minutes after oral administration. It has a bioavailability of approximately 100%.

Distribution

Caffeine diffuses rapidly into the extra vascular space. In the plasma it is weakly bound to circulating proteins (approximately 15%). It crosses the blood-brain barrier and its concentration in cerebrospinal fluid is the same as that in plasma.
Large amounts also pass into breastmilk which contain concentrations of 50% of those in plasma.

Metabolism and excretion

- *In adults:* caffeine is almost entirely metabolised in the liver by oxidation, demethylation, and acetylation.

- *In new-born infants:* metabolic capacity is reduced in new-born infants and caffeine is excreted in urine mostly in the unchanged form. It is eliminated far more slowly in new-born infants and has a half-life ranging between 36 and 144 hours.

Dosage adjustment

The ventilatory response correlates with plasma concentrations and the concentration range of 8 to 15 mg/L is accepted to be the therapeutic range. Adverse neurological and cardiovascular effects develop at plasma concentrations of 20 mg/L or over. Because of the large variability in kinetics in new-born infants, plasma concentrations monitoring, and dosage adjustments are required.

5.3. Preclinical safety data

Not completed.

6. PHARMACEUTICAL PARTICULARS

6.1. List of excipients

Water for injectable preparations

6.2. incompatibilities

In the absence of a compatibility study, this medicinal product must not be mixed with other medicinal products.

6.3. Shelf life

3 years.
After opening/reconstitution/dilution: the medicinal product must be used immediately.

6.4. Special precautions for storage

Store away from light.

6.5. Nature and contents of container

2 mL in type 1 clear glass ampoule.

6.6. Special precautions for disposal and other handling

No special requirements

7. MARKETING AUTHORISATION HOLDER

COOPERATION PHARMACEUTIQUE FRANCAISE
PLACE LUCIEN AUVERT
77020 MELUN CEDEX

8. MARKETING AUTHORISATION NUMBER(S)

34009 345 359 3 2: 2 mL in ampoule (clear glass), box of 10
34009 345 360 1 4: 2 mL in ampoule (clear glass), box of 50
34009 345 361 8 2: 2 mL in ampoule (clear glass), box of 100

9. DATE OF FIRST AUTHORISATION/RENEWAL OF THE AUTHORISATION

Date of first authorisation: 31 December 1997
Date of last renewal: 31 December 2012

10. DATE OF REVISION OF THE TEXT

03 May 2021

11. DOSIMETRY

Not applicable.

12. INSTRUCTIONS FOR PREPARATION OF RADIOPHARMACEUTICALS

Not applicable.

PRESCRIBING AND DISPENSING CONDITIONS

List I
