# Supplementary material for: The effect of an additional pre-extubational loading dose of caffeine citrate on mechanically ventilated preterm infants (NEOKOFF trial): Study protocol for a multicenter randomized clinical trial
Source: PLoS One. 2025 Jan 13;20(1):e0315856. doi: 10.1371/journal.pone.0315856 (PMC11730378; doi:10.1371/journal.pone.0315856)
Supplement: S4 File — (PDF) [file pone.0315856.s004.pdf]

A FORM

UNIT:

DOCTOR:

ADMISSION DATE AND TIME:

NEOKOFF NUMBER:

Questionnaire (A Form)

1. Patient personal details

First name: ____

Last name: ____

Mother's name: ____

Date of birth: ____

Gestational age: ____

Birth weight: ____

Gender:

□Female □Male □Not reported

2. Inclusion criteria

The preterm infant was born 32> gestational weeks : YES NO
The preterm infant had been mechanically ventilated for at least 48 hours: YES NO
The first attempted extubation : YES NO
Signed informed consent : YES NO
One „NO" is present = DO NOT INCLUDE!

3. Exclusion criteria

Major congenital anomailes : YES NO
The preterm infant had not recieved surfactant treatment: YES NO
Hydrops foetalis: YES NO
Persistent tachycardia before the extubation**: YES NO
Fetal/neonatal arrhytmia: YES NO
Asphyxia: YES NO
Lack of informed consent, refusal to participate in the study: YES NO
One „YES" is present = EXCLUDE!

**Persistent tachycardia: >200 bpm over 2 minutes

4. Details from the medical history

Antenatal steroid prophylaxis:
Complete:2 times Celestone, and 48h from the first dose or 4 times Dexamethasone, and 48h from the first dose. Incomplete: Delivery between 2 and 48 hours from the first dose. None: no antenatal steroid priphylaxis or the first dose was administered within 2 hours before birth.

□Complete □Incomplete □None

Mode of delivery:

□Sectio caesarea □Per vias naturales

Unit:

□Üllői □Bókay □Baross

Surfactant administration:

□One time □Repeated

Length of mechanical ventilation before extubation: _______________ hours

Type of mechanical ventilation:

□Conventional □HFOV □No data

Mean alveolar pressure before extubation: _______________ H2Ocm/mbar

Age at extubation: _______________ days

Systemic steroid use (for weaning from MV):

□Yes □For circulation or adrenal insuf. □No □No data

Mode of sedation:

□Continuous □Bolus □No sedation □No data

Mean heart rate (in the last 24 hours) prior to extubation: _______________ /min

Tachycardia during the previous 24 hours: _______________ %
(The time interval when the heart rate >200 (min) during the 24 hours before the extubation.)

Mean blood pressure (MAP) in the last 24 hours prior to extubation: _______________ Hgmm

Mean FiO2 (in the last 24 hours) prior to extubation: _______________ %

Last pH before extubation: _______________

Last pCO2 before extubation: _______________ Hgmm

Last glucose before extubation: _______________ mmol/L

Gastric residuals before the extubation: _______________ %
The mean value of gastric residuals 24 hours before the extubation. Measured 4 times/day, in percentage. Residual: x ml. Per os intake: y ml. Mean gastric residual is the mean value of the 4 percentage (x/y*100).

Eary-onset sepsis

□Positive blood culture □ CRP > 15 mg/L □Both □No □No data

Necrotizing enterocolitis before the extubation

□No □Stage I □Stage II □Stage III □No data

Intraventricular hemorrhage before the extubation

□No □Stage I □Stage II □Stage III □Stage IV □No data

Periventricular leukomalatia before extubation

□Yes □No □No data

Notes:

Assessed for eligibility but excluded from the study:

□Yes □No

Reason:

5. Randomization

The patient will be randomized by REDCap System in the following 2 arms:

Stratification is based on:

Gestational age < 28 weeks: □Yes □No

Antenatal steroide prophylaxis: □Yes □No

Yes if the first dose id given more than 2 hours before the preterm birth.

A. Interventional arm (A): 20 mg/kg pre-extubational loading dose of caffeine-citrate

B. Control arm(B): routine dosing of caffeine-citrate

RANDOMIZATION DATE AND TIME:

YEAR: ……………… MONTH: ……………. DAY: ……………… HOUR …………………. MIN: ……………………………………

ARM:

□A □B
