# Supplementary material for: The effect of an additional pre-extubational loading dose of caffeine citrate on mechanically ventilated preterm infants (NEOKOFF trial): Study protocol for a multicenter randomized clinical trial
Source: PLoS One. 2025 Jan 13;20(1):e0315856. doi: 10.1371/journal.pone.0315856 (PMC11730378; doi:10.1371/journal.pone.0315856)
Supplement: S5 File — (PDF) [file pone.0315856.s005.pdf]

B FORM

NAME:

NEOKOFF NUMBER:

Questionnaire (B Form)

1. Extubation

Time of extubation:

YEAR: MONTH: DAY: HOUR: MIN:

Type of non-invasive ventilation:

□ SNIPPV □DuoPAP/BiPAP □CPAP □Other □No data

NIV type if other: ____

PEEP: _______________H2Ocm/mbar

PIP: _______________H2Ocm/mbar

Repeating caffeine (5mg/kg) before the subsequent dose:

□Yes □No □No data

2. Details after extubation

Mean heart rate (in the first 24 hours) after extubation: _______________ /min

Tachycardia during the next 24 hours after extubation: _______________ %
(The time interval when the heart rate >200 (min) during the first 24 hours.)

Mean heart rate (in the second 24 hours) after extubation: _______________ /min

Tachycardia during the second 24 hours after extubation: _______________ %
(The time interval when the heart rate >200 (min) during the second 24 hours.)

Mean blood pressure (MAP) in the next 24 hours after extubation: _______________ Hgmm

Mean FiO2 (in the next 24 hours) after extubation: _______________ %

Time of the first blood gas after extubation:

YEAR: MONTH: DAY: HOUR: MIN:

Reintubation before the first blood gas**:**

□Yes □No

First pH after extubation: _______________

First pCO2 after extubation: _______________ Hgmm

B FORM

First glucose after extubation: _______________ mmol/L

Number of apneas within the next 24 hours after extubation: _______________
Desaturations (under 85%) with bradycardia (under 100 bpm); or desaturations that needs stimulation. In the next 24 hours.

Number of apneas within the second 24 hours after extubation: _______________
Desaturations (under 85%) with bradycardia (under 100 bpm); or desaturations that needs stimulation after extubation between the 24th and 48th post-extubational hour.

Reintuvation within the next 48 hours

□Yes □No

Time of reintubation:

YEAR: MONTH: DAY: HOUR: MIN:

Gastric residuals within the next 24 hours after before the extubation: _______________ %
The mean value of gastric residuals 24 hours before the extubation. Measured 4 times/day, in percentage. Residual: x ml. Per os intake: y ml. Mean gastric residual is the mean value of the 4 percentage (x/y*100).

Gastric residuals within the second 24 hours after before the extubation: _______________ %
The mean value of gastric residuals 24 hours before the extubation. Measured 4 times/day, in percentage. Residual: x ml. Per os intake: y ml. Mean gastric residual is the mean value of the 4 percentage (x/y*100).

Reduction of feeding

□Yes □No □No data

Cessation of feeding

□Yes □No □No data
