# Supplementary material for: The effect of an additional pre-extubational loading dose of caffeine citrate on mechanically ventilated preterm infants (NEOKOFF trial): Study protocol for a multicenter randomized clinical trial
Source: PLoS One. 2025 Jan 13;20(1):e0315856. doi: 10.1371/journal.pone.0315856 (PMC11730378; doi:10.1371/journal.pone.0315856)
Supplement: S6 File — (PDF) [file pone.0315856.s006.pdf]

C FORM

NAME:

NEOKOFF NUMBER:

Questionnaire (C Form)

1. Discharge assessement

Mechanical ventilation days during the length of hospital stay: _______________ days

NIV total days: _______________ days
NIPPV, CPAP, HFNC

Administration of loading dose of caffeine citrate in case of subsequent extubation:

□Yes □No □No data

Necrotizing enterocolitis after the extubation:

□No □Stage I □Stage II □Stage III □No data

Intraventricular hemorrhage after the extubation:
According to Papile's criteria. The highest stage should be chosen (e.g.: If there is a stage II on the left side, and Stage III on the right, Stage III should be chosen)

□No □Stage I □Stage II □Stage III □Stage IV □No data

Periventricular leukomalatia after extubation:

□Yes □No □No data

Late-onset sepsis:
Culture proven sepsis.

□Yes □No □No data

Patent ductus arteriosus:
Treatment was required (pharmacological or surgical).

□Yes □No □No data

Bronchopulmonary dysplasia:
Presence and stages of BPD at 36th weeks of postmenstural age. According to NICHD study 2019.

□Yes □No □No data

Death before discharge:

□Yes □No □No data

If died: Time of death:

YEAR: MONTH: DAY: HOUR MIN:

Any deviation from the protocol:

□Yes □No

Notes:
