# Supplementary material for: The effect of an additional pre-extubational loading dose of caffeine citrate on mechanically ventilated preterm infants (NEOKOFF trial): Study protocol for a multicenter randomized clinical trial
Source: PLoS One. 2025 Jan 13;20(1):e0315856. doi: 10.1371/journal.pone.0315856 (PMC11730378; doi:10.1371/journal.pone.0315856)
Supplement: S7 File — (PDF) [file pone.0315856.s007.pdf]

D FORM

NAME:

NEOKOFF NUMBER:

Questionnaire (D Form)

1. Follow up

Bayley score 1: ________________
Bayley score at one year of corrected age.

Bayley score 2: ________________
Bayley score at one year of corrected age.

Hearing impairment:
Based on documentation/specialist opinion

□Yes □No □No data

Visual impairment:
Based on documentation/specialist opinion.

□Yes □No □No data

Cerebral palsy:
Based on documentation/specialist opinion

□Yes □No □No data
