# Supplementary figures and images for: The effect of an additional pre-extubational loading dose of caffeine citrate on mechanically ventilated preterm infants (NEOKOFF trial): Study protocol for a multicenter randomized clinical trial
Source: PLoS One. 2025 Jan 13;20(1):e0315856. doi: 10.1371/journal.pone.0315856 (PMC11730378; doi:10.1371/journal.pone.0315856)

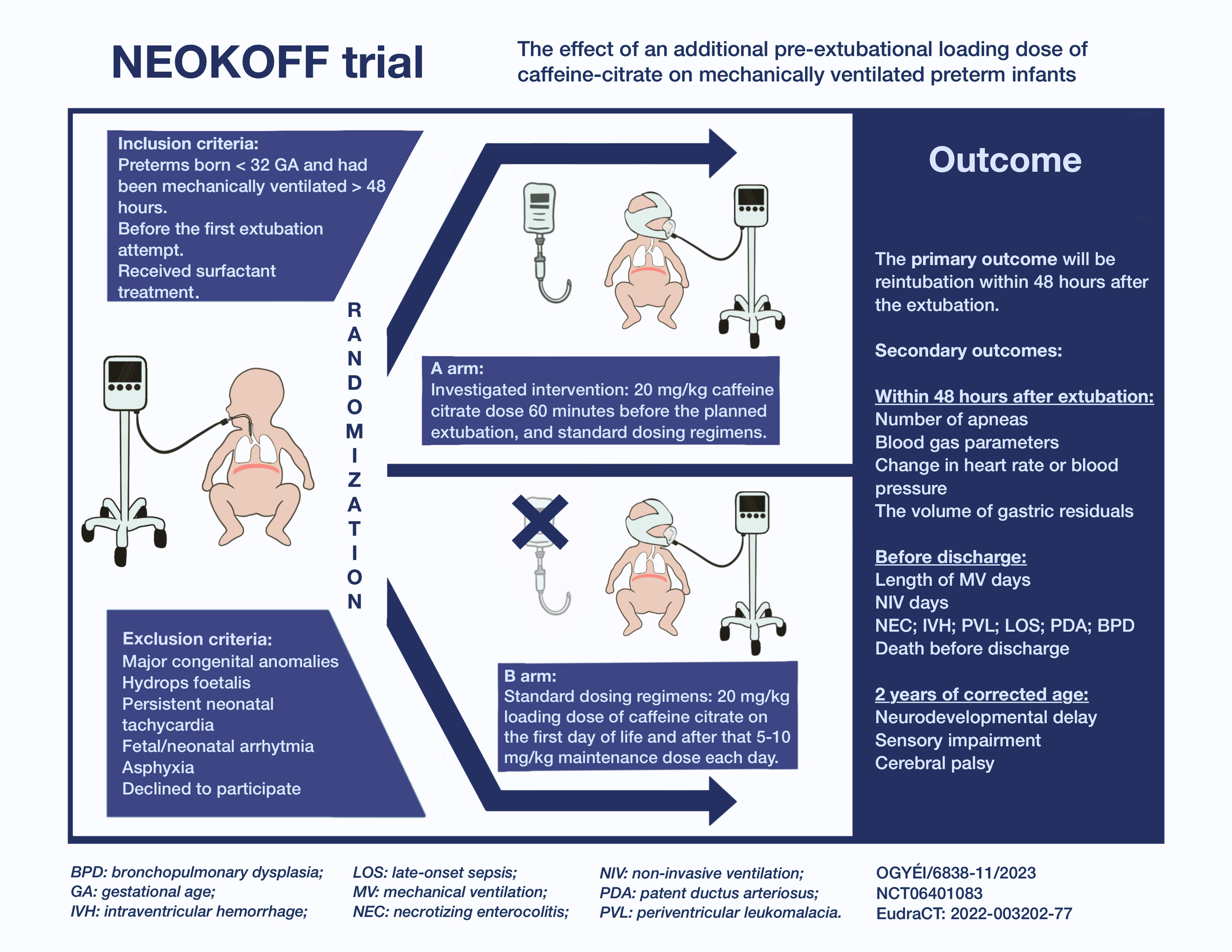

Supplement: S8 File — Republished from Graphical abstract for NEOKOFF randomized clinical trial under a CC BY license, with permission from Fatina Hanna JD, original copyright 2024. (TIF) [file pone.0315856.s008.tif]
