# Supplementary material for: The effect of an additional pre-extubational loading dose of caffeine citrate on mechanically ventilated preterm infants (NEOKOFF trial): Study protocol for a multicenter randomized clinical trial
Source: PLoS One. 2025 Jan 13;20(1):e0315856. doi: 10.1371/journal.pone.0315856 (PMC11730378; doi:10.1371/journal.pone.0315856)
Supplement: S9 File — (DOCX) [file pone.0315856.s009.docx]

**STUDY PROTOCOL**

**Study title**: The effect of an additional pre-extubational loading dose of caffeine citrate on extubational success rate

**Background:**

In Hungary, the rate of preterm births is higher than the EU average, hovering around 9% in recent years, according to data from the Central Statistical Office (KSH) [1]. Over the past decades, the survival rate of premature infants has significantly improved [2] [3]. Based on KSH data from 2017, the mortality rate of premature infants born with a birth weight below 1,000 grams has decreased to a quarter of the rate since 1990 [4]. Currently, one of the key goals in neonatology, alongside improving survival rates, is to reduce the incidence of complications among surviving preterm infants [5]. The immature lungs of preterm infants are susceptible to various damaging effects, such as oxygen toxicity and barotrauma or volutrauma caused by mechanical ventilation, leading to one of the most common complications of prematurity, bronchopulmonary dysplasia (BPD). Children with BPD are more likely to exhibit asthma-like symptoms and frequent upper respiratory tract infections during childhood. Lung function tests conducted in childhood have shown reduced lung function among children with BPD compared to their healthy peers [6].

Non-invasive respiratory support techniques have been promoted in neonatal care to reduce the incidence of BPD. Nevertheless, a portion of preterm infants still require mechanical ventilation during their care. One of the primary therapeutic goals when treating preterm infants is to minimize the duration of mechanical ventilation [7]. Although the goal is to achieve the earliest possible extubation, there are no consistent recommendations regarding the optimal timing and conditions. The frequency of extubation failure in preterm infants ranges between 10% and 80%. Optimizing the timing and conditions of extubation is crucial, as reintubation and repeated mechanical ventilation impose additional stress on preterm infants [8].

A common cause of the failure of non-invasive respiratory support is the weak spontaneous respiratory activity and recurrent apneic episodes of preterm infants. The apnea of prematurity has been widely studied in both domestic and international literature. Apnea is defined as a 15-20 second abruption of breathing or a shorter pause associated with bradycardia or desaturation. These pauses in breathing occur due to the immaturity of the respiratory center and chemoreceptors in preterm infants, as well as reduced patency of the upper airways [9]. Apnea is the most common indication for intubation and reintubation in preterm infants [10].

The stimulating effect of methylxanthines on the respiratory center and their apnea-reducing effect have been known for more than 40 years [11]. Based on current knowledge, caffeine is the first-line treatment for apnea. Among the methylxanthines, caffeine has the narrowest spectrum of side effects, the widest therapeutic range, and the longest half-life [9]. The 2007 Caffeine for Apnea of Prematurity (CAP) prospective, randomized clinical study, which examined 2000 preterm infants, found that caffeine reduced the incidence of BPD [12], improved survival without neurodevelopmental impairment [13], decreased the frequency of apnea, and facilitated weaning from mechanical ventilation [9].

Since the CAP study, the use of caffeine in preterm infants has become widespread worldwide. Caffeine is currently one of the most frequently used drugs in neonatal intensive care [11]. The most common dosing recommendation is a 20 mg loading dose of caffeine citrate followed by a maintenance dose of 5-10 mg daily [14]. Some studies have used higher loading and maintenance doses, suggesting that higher-dose caffeine treatment increases the rate of successful extubation [15-17]. However, other studies have reported more frequent side effects with higher doses [18, 19]. Based on conflicting literature data, caffeine dosing may vary by institution. Determining the optimal dose requires further basic and clinical research [20].

**Objectives**: We aim to answer whether a single loading dose of caffeine citrate before extubation affects the success of extubation. Furthermore, we intend to assess the frequency and severity of side effects. Our objectives include examining the impact of caffeine on the development of BPD, IVH, and PVL, as well as on the progression of IVH.

**Chief investigator: Ákos Gasparics MD, PhD**

**Investigators:** Kinga Kovács MD; Péter Varga MD, PhD; Leina Mahdi MD; Kata Szopori MD; Miklós Szabó DSc

**Setting:** Department of Obstetrics and Gynecology, Semmelweis University, Pediatric Center, Semmelweis University

**Design:**

Inclusion criteria are as follows:

- Preterm infants born before 32^nd^ week of gestation is completed;
- Were mechanically ventilated for at least 48 hours;
- Before the first planned extubation;

The exclusion criteria are:

- Lack of informed consent, refusal to participate in the study by parents or legal guardians;
- Major congenital anomaly;
- Hydrops foetalis;
- Had not received surfactant treatment
- Persistent tachycardia before extubation, fetal/neonatal arrhythmia;
- Asphyxia.

**Methods**:

The study is planned to include a total of 226 patients. In our units, preterm infants born before the 32nd gestational week receive standard dosing caffeine therapy. This involves a loading dose of 20 mg/kg on the first day of life, followed by a maintenance dose of 5-10 mg/kg caffeine citrate, administered intravenously once or twice daily.
Preterm infants who have been mechanically ventilated for at least 48 hours will be randomly allocated to intervention and control groups before the first planned extubation. Randomization will be conducted separately by each department, with stratification based on gestational age and steroid prophylaxis. The intervention is scheduled to take place 60 minutes before extubation.

- **“A” Arm:** 20 mg/kg loading dose (on the first day of life), followed by 5-10 mg/kg maintenance dose daily, and a 20 mg/kg loading dose administered slowly over 20 minutes infusion, 60 minutes before the planned extubation.
  - For birth weight under 1000 grams, diluted to 1 ml and administered at a rate of 3 ml/h.
  - For birth weight between 1000 and 2000 grams, diluted to 2 ml and administered at a rate of 6 ml/h.
  - For birth weight between 2000 and 3000 grams, diluted to 3 ml and administered at a rate of 9 ml/h.
- **“B” Arm:** 20 mg/kg loading dose (on the first day of life), followed by 5-10 mg/kg maintenance dose daily. Extubation will be performed according to the protocol of the respective department.

Data will be recorded during hospital treatment based on available documentation. Patient data will be collected using the data collection sheet provided in the appendix.
Given that the study is longitudinal, the patient’s personal data will also be recorded on the data collection sheet, necessary for future follow-up. The completed data collection sheets will be stored in a locked cabinet at the study site in compliance with current data protection regulations.

Independent of the data collection sheet, the workplace will maintain a Patient Identification Sheet, which, in addition to the code number, includes the patient’s name, date of birth, and social security number. Each workplace can only manage its data. Data processing and statistical analysis can be performed monthly. Existing documentation is stored electronically, encrypted, and password-protected. Only responsible personnel have access to the documentation.
The study will be conducted by neonatologists, pediatricians, residents, and nurses in the Neonatal Intensive Care Units.

**Patient Information and Consent:**

Before the start of the study, all guardians of the enrolled children will give written consent. The person providing the information will inform the guardian about the research's purpose and procedure using a brochure prepared by the research coordinator. The guardian will be informed that their consent to participate in the study is voluntary and can be withdrawn at any time, either orally or in writing, without any disadvantage. If the guardian withdraws consent, no clinical data related to their child will be used, even anonymously. The guardian can contact the research coordinator at any time with further questions.

**Implementation:**

- **A:** Collect data on premature infants born before the 32nd gestational week and treated in the Neonatal Intensive Care Units of the Department of Obstetrics and Gynecology/1st Department of Pediatrics, including name, gender, date of birth, and birth weight, inform parents about the study, and obtain consent.
- **B:** Assess eligibility for extubation (according to department protocol).
- **C:** Data collection: Average heart rate, average oxygen requirement, average mean airway pressure during the 24 hours before extubation; most recent blood gas analysis before extubation (pH; pCO2; glucose).
- **D:** Randomization before planned extubation. Implement intervention according to randomization.
- **E:** Extubation will be performed according to the department protocol, with a switch to non-invasive positive pressure ventilation (minimum PEEP: 6 cmH2O).
- **F:** Blood gas analysis will be performed after extubation according to department protocol (pH; pCO2; glucose), along with close monitoring for apnea and side effects. The primary outcome will be extubation failure, defined as reintubation within 48 hours. The criteria for reintubation will be based on the department protocol, and the exact time (in hours) from extubation will be recorded. We will also assess the average heart rate, oxygen requirement, and mean airway pressure during the 24 hours following extubation. Gastric residuals will be measured every 6 hours to assess gastric emptying,
- **G.** Cranial ultrasound within one week (IVH, PVL progression, or de novo)
- **H.** Assessement of BPD at 36^th^ week of postmenstrual age

**Statistical Methods**

The statistical analysis will be conducted using the R software package. Sample size calculations and the primary efficacy analysis are based on the expected extubation failure rate. We assumed that the extubation failure rate in the group receiving the loading dose ("A" arm) would be approximately 20%. In contrast, in the group not receiving the loading dose ("B" arm), the expected failure rate is 36.8%. Based on these assumptions, using the chi-square test, a sample size of 226 participants (113 per arm) has been calculated to provide 80% statistical power to detect a significant difference at a 5% significance level.

Secondary Safety Analyses: The safety outcomes will be evaluated using descriptive statistical methods. These will include the incidence of adverse effects and other complications observed during the study.

**Planned Study Timeline:**

The study is planned to be conducted from March 1, 2023, to January 01, 2028.

**Funding Sources:**

This study does not include additional costs. Any administrative expenses will be covered by the institutional budget.

**Publication Principles:**

The results of our study will be published anonymously in peer-reviewed journals with impact factors in both Hungarian and English and presented at national and international conferences focused on clinical and pediatric/neonatology topics.

**Budapest,**

**References:**

1. Hivatal, K.S., *Koraszülöttek és kis súlyú újszülöttek Magyarországon*. 2017, Központi Statisztikai Hivatal.

2. Varga, P., et al., *Survival and early complications of preterm infants with birthweight less than 500 grams during a 10-year period in Hungary.* Paediatr Perinat Epidemiol, 2020. **34**(5): p. 565-571.

3. Fathi, O., et al., *Development of a small baby unit to improve outcomes for the extremely premature infant.* Journal of Perinatology, 2022. **42**(2): p. 157-164.

4. Hivatal, K.S., *Csecsemőhalálozás*. 2019, Központi Statisztikai Hivatal: Budapest, Hungary.

5. Cheong, J.L.Y., et al., *Have outcomes following extremely preterm birth improved over time?* Seminars in Fetal and Neonatal Medicine, 2020. **25**(3): p. 101114.

6. Thébaud, B., et al., *Bronchopulmonary dysplasia.* Nature reviews. Disease primers, 2019. **5**(1): p. 78-78.

7. Al-Mandari, H., et al., *International survey on periextubation practices in extremely preterm infants.* Archives of Disease in Childhood - Fetal and Neonatal Edition, 2015. **100**(5): p. F428.

8. Sant'Anna, G.M. and M. Keszler, *Weaning infants from mechanical ventilation.* Clin Perinatol, 2012. **39**(3): p. 543-62.

9. Erickson, G., N.R. Dobson, and C.E. Hunt, *Immature control of breathing and apnea of prematurity: the known and unknown.* Journal of Perinatology, 2021. **41**(9): p. 2111-2123.

10. Bacci, S., et al., *Mechanical ventilation weaning practices in neonatal and pediatric ICUs in Brazil: the Weaning Survey-Brazil.* J Bras Pneumol, 2020. **46**(4): p. e20190005.

11. Kreutzer, K. and D. Bassler, *Caffeine for apnea of prematurity: a neonatal success story.* Neonatology, 2014. **105**(4): p. 332-6.

12. Schmidt, B., et al., *Caffeine therapy for apnea of prematurity.* N Engl J Med, 2006. **354**(20): p. 2112-21.

13. Schmidt, B., et al., *Long-term effects of caffeine therapy for apnea of prematurity.* N Engl J Med, 2007. **357**(19): p. 1893-902.

14. Eichenwald, E.C., *National and international guidelines for neonatal caffeine use: Are they evidenced-based?* Semin Fetal Neonatal Med, 2020. **25**(6): p. 101177.

15. Steer, P., et al., *High dose caffeine citrate for extubation of preterm infants: a randomised controlled trial.* Arch Dis Child Fetal Neonatal Ed, 2004. **89**(6): p. F499-503.

16. Chen, J., L. Jin, and X. Chen, *Efficacy and Safety of Different Maintenance Doses of Caffeine Citrate for Treatment of Apnea in Premature Infants: A Systematic Review and Meta-Analysis.* Biomed Res Int, 2018. **2018**: p. 9061234.

17. Moschino, L., et al., *Caffeine in preterm infants: where are we in 2020?* ERJ Open Research, 2020. **6**(1): p. 00330-2019.

18. Mohammed, S., et al., *High versus low-dose caffeine for apnea of prematurity: a randomized controlled trial.* European Journal of Pediatrics, 2015. **174**(7): p. 949-956.

19. McPherson, C., et al., *A pilot randomized trial of high-dose caffeine therapy in preterm infants.* Pediatr Res, 2015. **78**(2): p. 198-204.

20. Chavez, L. and E. Bancalari, *Caffeine: Some of the Evidence behind Its Use and Abuse in the Preterm Infant.* Neonatology, 2022.
