# Supplementary material for: The effect of an additional pre-extubational loading dose of caffeine citrate on mechanically ventilated preterm infants (NEOKOFF trial): Study protocol for a multicenter randomized clinical trial
Source: PLoS One. 2025 Jan 13;20(1):e0315856. doi: 10.1371/journal.pone.0315856 (PMC11730378; doi:10.1371/journal.pone.0315856)
Supplement: Attachment — Submitted filename: renamed_1016f.docx [file pone.0315856.s010.docx]

# COMMENTS – Manuscript PONE-D-24-29935

Title: “The effect of an additional pre-extubational loading dose of caffeine citrate on mechanically ventilated preterm infants (NEOKOFF trial): study protocol for a multicenter randomized clinical trial”

**Important note: This review pertains only to ‘statistical aspects’ of the study and so ‘clinical aspects’ [like medical importance, relevance of the study, ‘clinical significance and** **implication(s)’ of the whole study, etc.] are to be evaluated [should be assessed] separately/independently. Further please note that any ‘statistical review’ is generally done under the assumption that study specific methodological [as well as execution] issues are perfectly taken care of by the investigator(s). This review is not an exception to that and so does not cover clinical aspects {however, seldom comments are made only if those issues are intimately / scientifically related & intermingle with ‘statistical aspects’ of the study}. Agreed that** **‘statistical methods’ are used as just tools here, however, they are vital part of methodology [and so should be given due importance]. I look at the manuscript in/with statistical view point, other reviewer(s) look(s) at it with different angle so that in totality the review is very comprehensive. However, there should be efforts from authors side to improve (may be by taking clues from reviewer’s comments). Therefore, please do not limit the revision only (with respect) to comments made here.**

**COMMENTS:** Although there is no major flaw in the study/manuscript [and it is on very vital/important topic {namely “Extubation which involves removal of an endotracheal tube (ETT) from the trachea. Early and successful extubation can reduce complications such as ventilation acquired pneumonia (VAP)}], I have different opinion / observations/concerns or rather questions regarding a few issues which are given below:

Please refer to section on ‘Sample size’. As stated in line 119, “The calculated sample size is 226 patients (113 participants/arm)” is perfectly right, however, you need to adjust (inflate by 10% at least) the required sample size in the light of the fact that the proposed Chi-square test is ‘non-parametric’. Instead, one can/should apply continuity-corrected Z test and then the required sample size is Total 248 (124 in A, 124 in B). Moreover, note the Chi-square test is always two tailed [alternative hypothesis is two sided], whereas, you are interested in one sided alternative hypothesis (which may not matter much in this case, however, one should keep this thermotical point in mind. See the screen-shot below [from software ‘COMPRE2’ module of WIN-PEPI].

In line 202 you stated ‘’ drop-out rate is expected to be minimal due to the one-time intervention” seems to be incorrect [of doubtful value] as one-time intervention could be one of reasons of keeping the drop-out rate to the minimal level [it is not due]. Your claim perhaps sound diffently. As stated in lines 212-214 (Continuous variables will be compared using the t-test or Mann-Whitney test, whereas the χ2 test or Fisher’s exact test will be used for dichotomous outcomes.) following notes are pasted [in this context] from one famous standard textbook on ‘Medical Research Methodology’ {though I am sure that the authors already know these things}.

1. Though few variables are continuous in appearance they are likely to yield data that are in [at the most] ‘ordinal’ level of measurement [and not in ratio level of measurement for sure]. Then application of suitable non-parametric (or distribution free) test(s) is/are indicated/advisable then [even if distribution may be ‘Gaussian’ (also called ‘normal’)]. Agreed that there is/are no non-parametric test(s)/technique(s) available to be used as alternative in all situation(s), but should be used whenever/wherever they are available. Therefore, in short use suitable non-parametric test(s)/technique(s) while dealing with data that are in ‘ordinal’ level of measurement even if [despite that] the distribution may be ‘Gaussian’.

the Fisher’s exact test’ [which is commonly available in many software] is applicable to 2x2 tables only [for larger tables Exact Chi-square test is available – reference ‘A network algorithm for performing Fisher's exact test in r× c contingency tables’ Cyrus R Mehta, Nitin R Patel, Journal of the American Statistical Association, 1983, Volume: 78, Issue: 382, Pages 427-434].

Kindly provide more convincing explanation (give details) of the statement in lines 224-5: Due to the nature of the study population, participants do not need to be blinded and neither those performing the intervention nor treating clinicians will be blinded. Why you say that “participants do not need to be blinded” is not clear. You may be [most likely be] 100% correct (considering age & status of the subjects). What about ‘equipoise’ principle? Kindly remember [please excuse me for such a harsh comment/statement] that this is a scientific/academic document and so all details should be clearly/correctly communicated (do not take reader’s for granted).

It is appreciable that in line 47-8 you stated “Preterm infants in the control group will receive the standard dosing regimen” and later lines 142-3 that “The control group will receive the above-mentioned routine dosing regimens” as it is very important that the control group should receive the routine (present standard of care) dosing regimens [/ active controls] in such trials. I suggest to mention this fact [handling of the control group] more prominently (as these are the only two occasions).

Good that Supporting information includes [S1 File:] SPIRIT checklist. But referring to CONSORT guidelines is also desirable. It is well-known that while reporting {findings from and even planning} ‘Clinical Trial’ one should follow CONSORT guidelines. Since your article type is protocol of ‘Clinical Trial’, you are supposed to cover these items in the report or even in ‘Protocol’ (even if you may not use them)]. Surprisingly, any information is not proposed to be displayed in tables. All important information is planned to be included only as ‘Supporting information’ and not in the main text [highly desirable].

Limitations (if any) of the study are not mentioned/listed anywhere. Does that mean {according to authors} there are none? As pointed out in ‘important note’ above “This review pertains only to ‘statistical aspects’ of the study and so ‘clinical aspects’ should be assessed separately/independently. In my opinion, to make this article more (completely) acceptable (which is quite possible and easy), a small amount of re-vision (re-drafting) may be needed. ‘Minor revision’ is recommended.
