# Supplementary material for: The effect of an additional pre-extubational loading dose of caffeine citrate on mechanically ventilated preterm infants (NEOKOFF trial): Study protocol for a multicenter randomized clinical trial
Source: PLoS One. 2025 Jan 13;20(1):e0315856. doi: 10.1371/journal.pone.0315856 (PMC11730378; doi:10.1371/journal.pone.0315856)
Supplement: Attachment — Submitted filename: review_10.24.docx [file pone.0315856.s011.docx]

I had the pleasure to review the article titled “The effect of an additional pre-extubational loading dose of caffeine citrate on mechanically ventilated preterm infants (NEOKOFF trial): study protocol for a multicenter randomized clinical trials”.

The authors aim to investigate whether administering an additional loading dose of caffeine citrate one hour prior to extubation improves the success of extubation. The article is presented as a multicenter randomized clinical trial **protocol.**

Abstract:

- Aim: The specific aim or objective of the study should be explicitly stated in the abstract to clarify the central research question.

Methods:

- Inclusion Criteria: It would be helpful to specify whether there is a threshold for chronological age among the participants.
- Randomization: The randomization procedure mentions an allocation ratio of 1:1, but it would be beneficial to provide more clarity on how this will be achieved or operationalized within the trial.
